# Eye tracking assessment of Parkinson's disease: a clinical retrospective analysis

**DOI:** 10.25122/jml-2024-0270

**Published:** 2024-03

**Authors:** Emanuel Ştefănescu, Ştefan Strilciuc, Vlad-Florin Chelaru, Diana Chira, Dafin Mureşanu

**Affiliations:** 1 Department of Neuroscience, Iuliu Hațieganu University of Medicine and Pharmacy, Cluj-Napoca, Romania; 2 RoNeuro Institute for Neurological Research and Diagnostic, Cluj-Napoca, Romania; 3 Research Center for Functional Genomics, Biomedicine, and Translational Medicine, Iuliu Hațieganu University of Medicine and Pharmacy, Cluj-Napoca, Romania

**Keywords:** eye-tracking, Parkinson’s disease, visually guided saccades

## Abstract

Parkinson's disease (PD) presents a significant clinical challenge due to its profound motor and cognitive impacts. Early diagnosis is crucial for implementing effective, stage-based treatment strategies. Recently, eye-tracking technology has emerged as a promising tool for the non-invasive diagnosis and monitoring of various neurological disorders, including PD. This retrospective study analyzed eye-tracking parameters, specifically visually-guided saccades (VGS), in PD patients within a clinical setting. We reviewed eye-tracking data from 62 PD patients, focusing on eye movement performance in horizontal and vertical VGS tasks. Our findings revealed significant correlations between demographic profiles, Mini-Mental State Examination (MMSE) scores, pattern recognition, and spatial working memory tests with saccadic performance in PD patients. Despite the retrospective nature of the study, our results support the potential of eye-tracking technology as a valuable diagnostic tool in PD assessment and monitoring. Future research should prioritize longitudinal studies and more comprehensive assessments to further understand and enhance the clinical application of eye-tracking in PD.

## INTRODUCTION

Parkinson's disease (PD) is a complex and progressive neurological disorder characterized by motor and cognitive impairments. The etiology of PD is considered to result from a dynamic interplay of genetic and environmental factors affecting fundamental cellular processes. Diagnosing PD primarily relies on clinical evaluation, which assesses motor symptoms like bradykinesia, rigidity, and resting tremors, with postural instability indicating advanced stages [1–3].

The progression of PD can be divided into three phases: preclinical (neurodegeneration has started, but no symptoms are evident), prodromal (symptoms are present but insufficient for a definitive diagnosis), and clinical (diagnosis based on classical motor signs). Many markers for prodromal PD are not well-validated, and their sensitivity and specificity vary, making early diagnosis challenging [4,5].

Eye tracking provides a valuable tool for diagnosing neurological disorders non-invasively and with high precision. It assists with detecting and monitoring these conditions at an early stage by using quantitative metrics. It provides a valuable addition to conventional assessments and can be used for various conditions. [6–8]. Eye movement abnormalities in PD patients include hypometric saccades, frequent square wave jerks, impaired inhibition of reflexive saccades, poor visual convergence ability, impaired smooth pursuit, increased latency of voluntary saccades, and ocular tremor. These abnormalities lead to less accurate, multi-step, or broken-down saccades. Patients with PD experience difficulties with convergence, affecting their focus on near objects, and exhibit altered smooth pursuit, making tracking moving objects challenging. These eye movement issues impair daily activities like visual search, reading, and walking stability [9–12].

The primary aim of this retrospective study was to analyze the relationship between visually guided saccadic (VGS) parameters, demographic variables, and cognitive function in patients with PD within a clinical setting. By doing so, we seek to contribute to the current literature on eye-tracking as a reliable, non-invasive tool for the clinical assessment and monitoring of PD progression.

## MATERIAL AND METHODS

This observational, retrospective study was conducted as part of the doctoral study titled: “Developing a new standard for assessing brain injury using eye tracking" at Iuliu Hațieganu University of Medicine and Pharmacy. The experimental procedure was conducted in conformity with the Declaration of Helsinki. The evaluation of subjects included in this retrospective study is based exclusively on analyzing existing data without involving any direct interventions or gathering new data.

Sixty-two patients with a documented diagnosis of PD were included in this study. The research aimed to evaluate the characteristics of eye movements and the potential of eye-tracking technology as a diagnostic tool for PD in a clinical setting. The accessible population comprised patients who presented at the RoNeuro Institute for Neurological Research and Diagnostic, Cluj-Napoca, Romania and underwent clinical, psychological, and eye-tracking evaluations between January 2016 and December 2022.

We excluded patients with severe systemic disorders or terminal malignancy, severe psychiatric disorders, acute or advanced chronic ophthalmological disorders, or a history of stroke or traumatic brain injury before data collection. Additionally, individuals who completed neurological evaluations but did not perform eye-tracking assessments were removed from this analysis.

### Study procedure

Each 62 patients with PD underwent a thorough clinical neurological examination by a trained physician. Available psychological evaluations were conducted by a certified psychologist.

Horizontal and vertical VGS were assessed over approximately 10 minutes using the Tobii Tx300 eye-tracking system (Tobii Technology, Stockholm, Sweden). The visual stimulus sequence was created with Tobii Studio 3.4.8 software and displayed on a 23-inch screen with a 16:9 aspect ratio, 1920x1080 pixel resolution, 60Hz refresh rate, and a 5 ms response time. The system provided an average gaze accuracy of 0.4° and a processing latency between 1.0 and 3.3 ms [13,14].

Each eye movement recording session began with a briefing on the task requirements and continued with a session of practice trials. Tobii TX300 calibration procedure, using a 9-point system, was performed before each eye tracking task. Binocular gaze data was collected at a 250Hz sample rate. The experiment occurred in a quiet, dimly lit room to limit external influences on gaze behavior. Participants were positioned 65 cm from the screen and seated in a comfortable, adjustable chair with a chin and forehead rest to restrict head movement. The visual stimulus consisted of a black background (luminance of 2.5 cd/m^2^) and a red dot with a diameter of 0.4° (luminance of 63 cd/m^2^) [15].

Every horizontal saccadic trial started with a 1500 ms presentation of a central dot target, followed by a 200 ms interval without any stimulus (gap period). Subsequently, a dot target was displayed at a specific eccentric horizontal location for 1500 ms (+/-10 and +/-18° visual angles). The trial timing was identical for the vertical saccadic task, while the central dot shifted position upward and downward at 8° of visual angle along the central vertical axis. To avoid predictability, the appearance of target stimuli was randomized. Participants were instructed to fixate on the red target throughout the experiment. To ensure a sufficient sample size for further analysis, our protocols incorporated a total of 40 stimulus presentations for each task, ten for each tested amplitude [16–18].

The Tobii IV-T fixation filter was applied with the following parameters: eye selection based on the average data from both eyes, velocity window length was set at 20 ms, gap-fill interpolation at 75 ms, all adjacent fixations were merged if the in-between interval was ≤75 ms and the angular distance was ≤0.5°, minimum fixation duration was set at 60 ms, with the I-VT classifier threshold at 30°/s, and noise reduction was disabled. Exported recordings from Tobii Studio were further analyzed using an in-house-built platform that automatically determines saccadic parameters and allows a visual check of eye movements performed during the recording [19].

We applied additional validity criteria for trials to guarantee the accurate perception of the offset of the central fixation dot. Trials were considered valid if no saccadic eye movements or blinks were detected within 60 ms before the disappearance of the central dot. Furthermore, the gaze of the subject needed to be within a 100-pixel (approximately 2.3°) radius from the center of the screen, defined as the starting point. Only the first saccade originating from this starting point was included after the eccentric target onset. Saccades with a latency of less than 100 ms were classified as anticipatory and excluded from analysis [16,17]. Trials were classified based on the target's eccentricity: near and far for horizontal trials and up and down for vertical trials. Only subjects with a minimum of 6 valid trials were included in the study. A description of saccadic parameters calculated for each VGS is avaiable in Table 1. The blink rate (number of blinks per minute) during the VGS tasks was also determined [20]. Blinks were detected from the raw data as intervals when gaze tracking data indicated 0 (high confidence) for the validity of left and right eye identification [14].

**Table 1:** Parameters calculated for each VGS

Saccadic parameter	Definition	Unit
Latency	The time interval between the appearance of the eccentric dot and the start of the VGS	ms
Duration	The time interval difference between the start and the end of VGS	ms
Amplitude	The difference in degrees of visual angle between gaze location at the start and the end of the VGS	°
Mean velocity	The average velocity of the VGS	°/ms
Peak velocity	Maximum velocity of the VGS	°/ms
Gain	The ratio between amplitude saccade/amplitude target	

We collected demographic and clinical data for all 62 patients with PD from the RoNeuro Institute database. This data included age, sex, primary neurological diagnosis, and PD stage per the Hoehn & Yahr scale. Additionally, where psychological evaluations were available, we extracted scores from the Mini-Mental State Examination (MMSE) and results from two Cambridge Neuropsychological Test Automated Battery (CANTAB) tests: Pattern Recognition Memory (PRM) and Spatial Working Memory (SWM). The MMSE is a widely utilized tool for rapid cognitive assessment, especially in hospitalized and elderly patients [21]. The PRM task, a two-choice forced discrimination test, evaluates visual pattern recognition memory [22,23]. The CANTAB SWM test assesses spatial memory by challenging subjects to remember the location of items in an array and then recall those locations after a brief delay. This test provides valuable insights into an individual's ability to hold and manipulate spatial information [22].

Psychological evaluations were not identified for all patients included in the present study. We extracted only 42 MMSE evaluations and 22 CANTAB evaluations. Where available, we included the following psychological evaluation variables: MMSE total score from CANTAB tests – PRM - Percent Correct Delayed (CANTAB_PRMCD), PRM - Mean Correct Latency Delayed (CANTAB_PRMMCLD) – measured in ms, PRM – Percent Correct Immediate (CANTAB_PRMPCI), PRM – Mean Correct Latency Immediate (CANTAB_PRMMCLI) – measured in ms, SWM – Total Errors (CANTAB_SWM_TE).

### Statistical analysis

For our statistical analyses, we utilized R version 4.3.2 with RStudio, employing several libraries: 'openxlsx' for spreadsheet file interaction and 'ggplot' along with 'patchwork' for creating graphical representations. We conducted all analyses with a significance threshold set at alpha = 0.05. Pearson and Spearman correlation analyses were carried out to explore the relationships between demographic variables, neuropsychological scores, and VGS task parameters [24–28].

## RESULTS

**Figure 1:**
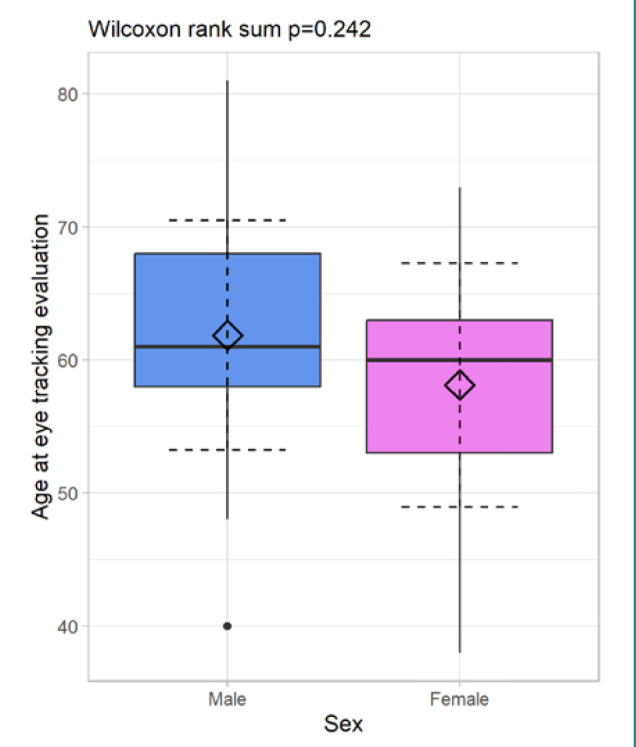
Distribution of patient age at eye tracking evaluation by gender

Our study included an eye-tracking evaluation of VGS for 62 patients clinically diagnosed with PD. We analyzed recordings of 37 male (59.68%) and 25 female (40.32%) patients. Age distribution is reported in Table 2 and Figure 1.

**Table 2:** Age of patients at eye tracking evaluation

	Mean	Median	SD
All subjects	60.35	60.5	8.98
Female	58.12	60	9.18
Male	61.86	61	8.64

**Figure 2:**
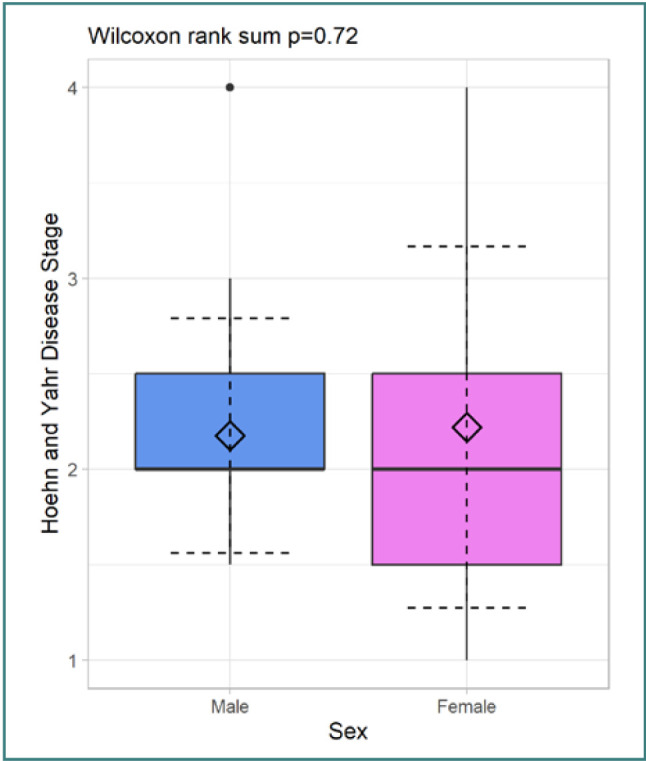
Distribution of Hoehn and Yahr disease stage by gender

To further understand the patient demographics, we also examined the distribution of the Hoehn and Yahr stages among the patients, which categorize the severity of the disease. The average HY stage was 2.17 for male and 3.2 for female patients. Table 3 and Figure 2 present the distribution of patients with PD based on the Hoehn and Yahr stages. Psychological evaluation scores distribution is reported in Tables 4 and 5.

**Table 3:** Hoehn & Yahr distribution among the study population

	Hoehn & Yahr staging of patients with PD					
**Stage**	**1**	**1.5**	**2**	**2.5**	**3**	**4**
**Total**	*n* = 3 (4.84%)	*n* = 12 (19.35%)	*n* = 29 (46.77%)	*n* = 6 (9.68%)	*n* = 6 (9.68%)	*n* = 6 (9.68%)
**Male**	*n* = 0 (0%)	*n* = 7 (11.29%)	*n* = 20 (32.26%)	*n* = 4 (6.45%)	*n* = 4 (6.45%)	*n* = 2 (3.23%)
**Female**	*n* = 3 (4.84%)	*n* = 5 (8.06%)	*n* = 9 (14.52%)	*n* = 2 (3.23%)	*n* = 2 (3.23%)	*n* = 4 (6.45%)

**Table 4:** Distribution of MMSE scores among 42 patients

	Mean	SD
Overall	28.0	2.61
Female	28.6	1.82
Male	27.62	3.06

**Table 5:** Distribution of CANTAB scores among 22 patients

	CANTAB_PRMCD	CANTAB_PRMMCLD	CANTAB_PRMPCI	CANTAB_PRMMCLI	CANTAB_SWM_TE
**Mean**	0.77	2631.86	8.36	2529.89	23.23
**SD**	0.12	560.96	24.33	652.80	8.33

### Age-related correlations

**Figure 3:**
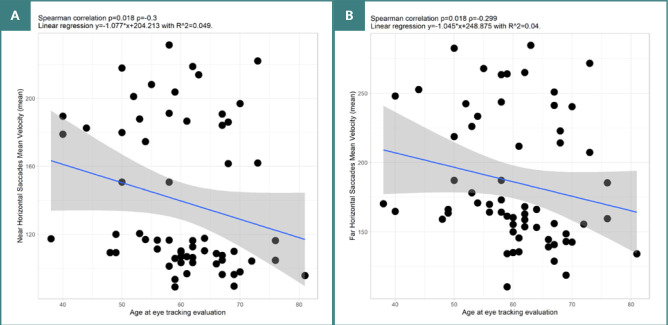
Spearman correlation between age of patients with PD at eye tracking evaluation and VGS parameters. A, Correlation between age and mean velocity of near horizontal VGS. B, Correlation between age and mean velocity of far horizontal VGS.

Our analysis revealed several significant correlations between age and various parameters of horizontal VGS. For near-target VGS, there was a negative, weak correlation between age and amplitude (*P* = 0.027, ρ = -0.281). Significant moderate negative correlations were also observed between age and mean velocity (*P* = 0.018, ρ = -0.3) (Figure 3), as well as with gain (*P* = 0.032, ρ = -0.272). The results remained partly consistent for more eccentric targets. Significant negative, weak correlations were found between age and mean velocity (*P* = 0.018, ρ = -0.299). Furthermore, for VGS to more eccentric targets, there was a significant positive moderate correlation between age and latency (*P* = 0.005, ρ = 0.353).

**Figure 4:**
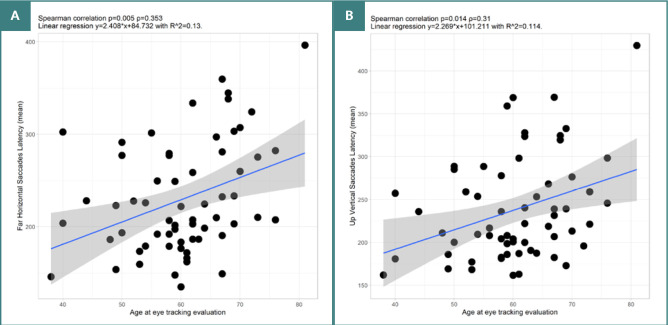
Spearman correlation between age of patients with PD at eye tracking evaluation and VGS parameters. A, Correlation between age and latency of far horizontal VGS. B, Correlation between age and latency of upward vertical VGS.

As for upward vertical VGS, we identified a significant positive moderate correlation between age and latency (*P* = 0.014, ρ = 0.31) – see Figure 4.

### MMSE

**Figure 5:**
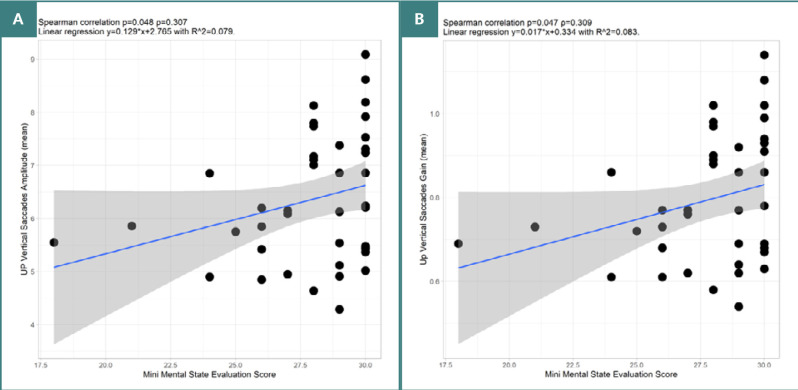
Spearman correlation between MMSE score and vertical VGS parameters. A, Correlation between MMSE score and amplitude of upward vertical VGS. B, Correlation between MMSE score and gain of upward VGS.

MMSE scores correlated with parameters of vertical VGS made upwards. Significant positive moderate correlations were observed with amplitude (*P* = 0.048, ρ = 0.307) and gain (*P* = 0.047, ρ = 0.309), Figure 5.

### CANTAB

For patients who underwent evaluation with the PRM task, the variable CANTAB_PRMCD showed a positive moderate correlation with the percentage of valid vertical VGS (*P* = 0.03, ρ = 0.464).

**Figure 6:**
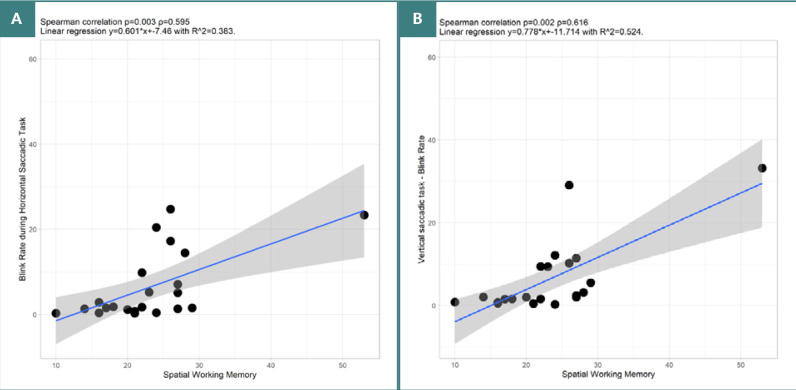
Spearman correlation between CANTAB – SWM and Blink Rate. A, SWM, and blink rate during horizontal VGS. B, SWM, and blink rate during vertical VGS.

SWM test scores showed several notable correlations with VGS parameters. There was a significant positive high correlation between CANTAB_SWM_TE and blink rate for both horizontal VGS (*P* = 0.003, ρ = 0.595) and vertical VGS (*P* = 0.002, ρ = 0.616) Significant positive high correlations were also found between SWM and duration of VGS irrespective of target location (*P* = 0.003, ρ = 0.6 and *P* = 0.015, ρ = 0.5090) (Figure 6). Furthermore, there was a significant negative correlation between CANTAB_SWM_TE and the latency of upward VGS (*P* = 0.035, ρ = -0.452).

No significant correlations were identified between the Hoehn and Yahr stage of PD or disease duration and any eye-tracking parameters derived from horizontal and vertical VGS tasks.

## DISCUSSION

Our study investigated the potential of eye-tracking technology as a diagnostic tool for PD by analysing VGS parameters in 62 patients clinically diagnosed with PD. Significant correlations were observed between various saccadic outcome variables, patients' age, and neuropsychological evaluations.

### Age-related changes in horizontal and vertical VGS

In the present study, we found a negative correlation between the age of the patients and amplitude, mean velocity, and gain of horizontal VGS, largely independent of the eccentricity of the presented target. These outcomes align with current findings in eye movement research. Saccade amplitude tends to decrease with age, a trend observed in both natural aging and in patients with PD, where saccadic amplitudes are progressively lower across different stages of cognitive impairment [29]. In advanced stages of PD, the reduction in saccadic amplitude is more acute, indicating that disease progression, in conjunction with age, significantly impacts saccadic amplitude [29,30]. As for the influence of age on saccadic velocity profile, current perspectives on saccadic velocities do not show a significant difference between elderly controls and PD groups, suggesting that within an elderly sample, age does not significantly influence saccadic velocity [29]. However, peak saccadic velocities are significantly reduced in patients with advanced PD, highlighting that disease severity rather than age alone is a critical factor [29,30]. This reduction in velocity, particularly prevalent in advanced stages compared to mild stages, may be attributed to improper coactivation of opposing ocular muscles, which becomes more evident with disease progression [31].

Factors such as age or PD may affect the gain of horizontal saccades. However, while there is extensive research on the changes in amplitude and velocity of saccades with age, the specific age-related changes in gain are not equally documented [29,31].

Older subjects showed increased delays in initiating horizontal VGS eye movements to more eccentric targets and upward vertical locations. This indicates that with advancing age, these patients are faced with a decline in the accuracy and speed of their eye movements and longer times to plan and initiate eye movements toward upward locations and to eccentric locations in the visual field. Some studies show that age is associated with prolonged saccadic latency, particularly in individuals with cognitive impairments such as PD-MCI (Parkinson's disease mild cognitive impairment) and PDD (Parkinson's disease dementia)[30]. On the other hand, it has been shown that patients with PD have increased latencies in vertical VGS and antisaccades (AS) compared to healthy controls. This increase in latency is a significant marker of motor and cognitive impairment in PD [12,31]. Moreover, Levodopa, a common medication for PD, prolongs the latency of vertical VGS, but we did not account for the medication received [31].

### Cognitive evaluation outcomes and eye-tracking metrics

Cognitive function is a complex, ever-changing process affected by the interaction of multiple systems and subsystems. It involves transforming and utilizing sensory input, coordinating sub-processes like memory and attention, and is modulated by neural connectivity, metacognition, and mind-wandering. Homeostatic regulation ensures equilibrium and optimal functioning [32]. Our subgroup analysis of the 42 patients evaluated with MMSE showed notable correlations between cognitive measurements and eye-tracking metrics. Greater cognitive abilities were associated with the amplitude and precision (gain) of the vertical VGS directed to an upward target, similar to current PD research [33,34].

In the 22 patients who underwent two CANTAB neuropsychological evaluations, we found a significant moderate positive correlation between the percentage of correctly selected patterns in the delayed forced-choice condition of the PRM task and the percentage of valid vertical VGS made towards upward targets. This suggests a potential relationship between visuo-spatial memory performance and the capacity to perform vertical eye movements in patients with PD, indicating shared neural mechanisms or pathways. Patients with PD experience deficits in visuospatial memory, essential in tasks requiring the retention and manipulation of spatial information. They show impairments in planning and executing such tasks showing frontal lobe and basal ganglia dysfunction. This dysfunction of the prefrontal-basal ganglia circuit seen in PD also affects the suppression of automatic saccades. The severity of PD correlates with greater impairments in visuo-spatial memory and vertical eye movement [35–41].

SWM test outcomes revealed several significant correlations with eye-tracking metrics. Noteworthy, there was a strong positive correlation between blink rate and the duration of both horizontal and vertical VGS for both near and far targets. A higher number of errors in the SWM test was associated with an increased blink rate and longer duration of horizontal VGS. PD significantly impacts the blink rate and kinematic properties of blinking. Patients with PD have a lower blink rate compared to healthy individuals, with considerable disparity among patients. Kinematic properties of blinking, such as blink duration, amplitude, and peak velocity, are altered in patients experiencing PD. It has been observed that, in PD, blinks have shorter durations, smaller amplitudes, and longer pauses between phases. Small blink waves before blink onset are prevalent in patients with PD. Although disease severity exacerbates these impairments, current research did not show a significant correlation with clinical metrics like the Hoehn-Yahr score [42,43]. Moreover, patients with PD face reduced central dopaminergic activity associated with decreased blink rates and impairments in cognitive functions, including working memory [44]. Interestingly, individuals with PD who make more errors in the SWM task tend to have higher blink rates during the VGS task. This implies that the level of cognitive load and task difficulty can influence the variability in blink rate. Even though patients with PD typically exhibit reduced spontaneous blink rates, their blink rate may increase during challenging tasks in response to cognitive and motor demands.

Lastly, the difficulties in the SWM task showed a moderate negative correlation with the latency of upward VGS, indicating that impairments in spatial working memory were linked to shorter latency, possibly affecting the capacity to inhibit anticipatory eye movements towards upward targets.

Our study has several limitations. As a retrospective, observational study, our findings are inherently constrained by the type of data collected and may introduce selection bias. The small sample size (*n* = 62) and specific exclusion criteria applied may affect the generalizability of our results.

The study also lacked long-term follow-up, limiting our ability to observe longitudinal changes in saccadic parameters over time and their relationship with disease progression. The relationship between eye-tracking metrics and the severity of PD is limited to the Hoehn and Yahr scale. Lastly, eye tracking evaluation relied only on horizontal and vertical VGS tasks.

## CONCLUSION

Our study highlights the potential of eye-tracking technology in diagnosing Parkinson's disease by analyzing VGS parameters in 62 patients clinically diagnosed with PD. Age-related declines were observed, with older patients showing impairments in performing horizontal and vertical VGS. Higher MMSE scores correlated with the precision of vertical saccades. Increased visuospatial memory capabilities in correctly selecting patterns were associated with a higher percentage of valid vertical saccades, while spatial working memory test outcomes showed significant correlations with eye tracking metrics such as blink rate and saccade duration. Despite these limitations, our data support eye tracking as a non-invasive diagnostic tool. Future research should focus on longitudinal studies, larger and more homogenous cohorts, the use of eye-tracking systems with higher sample rates and a more diverse battery of tests, including visual search performance tasks, and comprehensive assessments of disease severity and progression like the Unified Parkinson's Disease Rating Scale (UPDRS) to enhance understanding and application in PD evaluation.
